# Selumetinib produces a central core of apoptosis in breast cancer bone metastases in mice

**DOI:** 10.18632/oncoscience.102

**Published:** 2014-11-27

**Authors:** Nicholas A. Bosma, Arvind K. Singla, Charlene M. Downey, Frank R. Jirik

**Affiliations:** ^1^ Department of Biochemistry and Molecular Biology, The McCaig Institute for Bone and Joint Health University of Calgary, Calgary, Alberta, Canada

**Keywords:** bone metastases, AZD6244 (Selumetinib), activated KRAS, triple-negative breast cancer, bioluminescence imaging, apoptosis

## Abstract

Bone is a common site for metastatic colonization in patients with breast cancer, hence the importance of identifying new treatments for this disease. Preclinical studies of bone metastases have commonly employed MDA-MB-231 cells that possess an activated *KRAS* allele. While activating *RAS* mutations are relatively rare in human breast cancer, increased RAS-RAF-MEK pathway

activity is common in high-grade breast cancers. To study the consequences of MEK inhibition on bone metastases stemming from the intra-cardiac injection of luciferase-expressing MDA-MB-231 cells in mice, we used the MEK inhibitor AZD6244 (Selumetinib). We found that AZD6244 treatment caused decreased tumor bioluminescence that was associated with cavitation of the bone metastases, owing to apoptosis of cells specifically within the central region of the bone lesions. Hypothesizing that the latter effect was due to the increased sensitivity of poorly perfused regions to pro-apoptotic stimuli, we found that the combination of serum deprivation and AZD6244 led to dramatic induction pf MDA-MB-231 apoptosis *in vitro*. Our results suggest that MEK inhibition may be a strategy for triggering cell death within the hypoperfused, oxygen and nutrient poor regions of tumors with activated *RAS* alleles.

## INTRODUCTION

Bone is a frequent site of metastatic colonization in advanced breast cancer, occurring in up to 70% of individuals with this disease [1]. Once tumors have metastasized to bone, 5 year survival rates are in the order of 20% [1, 2]. Skeletal metastases represent a relatively difficult target for therapeutic intervention, owing in part to the complex and supportive nature of the stromal microenvironment that acts to enhance tumor cell viability and growth. Current treatment options are limited and largely palliative, and include radiation therapy, chemotherapy, and bisphosphonate administration [3, 4]. With respect to the latter, for example, factors elaborated by breast cancer cells may be able inhibit bisphosphonate-induced osteoclast apoptosis, ultimately limiting the effectiveness of this front-line treatment [5]. Thus, there remains a need for additional treatments able to target breast cancer metastases within the bone microenvironment.

Mutational activation of *RAS* family genes, seen in a large percentage of all human malignancies [6], makes it one of the most frequent pro-oncogenic drivers in diverse tumor types, including breast cancer. Although activating *RAS* mutations are most frequently found in cancers arising in the colon, lung, pancreas, and thyroid [7], they are nevertheless seen in about 5% of breast malignancies [8]. The relative rarity of activating *RAS* mutations in breast cancer has led to the notion that the RAS signal transduction pathway activity does not play an important pathogenic role in this disease. However, a large proportion of individuals with breast cancer show over-expression of EGFR, a kinase whose activation leads to RAS pathway activation. Indeed, 70-80% of breast carcinomas demonstrate evidence of EGFR over-expression [9], and this may in part be responsible for the RAS pathway activation observed in breast cancer [10]. Furthermore, RAS pathway activation has been implicated in breast cancer invasion and growth [8], as well as in mediating resistance to chemotherapy [11]. Therefore, targeting of specific RAS downstream components holds the potential to be effective against a range of different tumor types, including osteolytic breast cancer metastasis, as we investigated herein via the use of a small molecule inhibitor that targets MEK.

In this study we utilized a well-established model of breast cancer bone metastasis that mimics the processes involved in the metastatic colonization process, including extravasation, colonization of suitable marrow microenvironments [12-16], and induction of extensive osteolytic damage as a result of osteoclast activation. We examined the effect of AZD6244 (Selumetinib) on both nascent as well as established skeletal metastasis stemming from the intracardiac injection of the human breast cancer-derived cell line, MDA-MB-231, that harbors an activating mutation of *KRAS*.

*In vivo* bioluminescence imaging revealed that AZD6244 treatment dramatically inhibited the growth of luciferase-expressing MDA-MB-231 bone metastases and led to central cavitation of these lesions.

## RESULTS

### AZD6244 inhibits growth of osteolytic metastases

**Figure 1:**
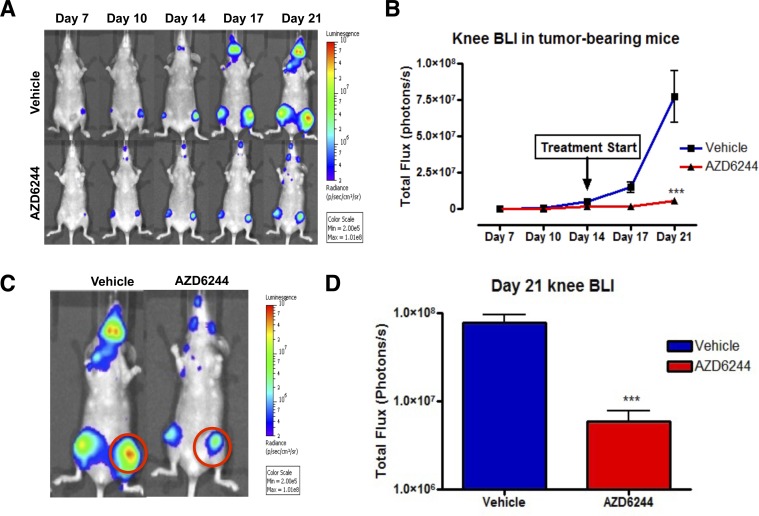
AZD6244 treatment slowed the growth of bone metastases (A) Bioliuminescence imaging (BLI; ventral image) of mice following intracardiac injection of MDA-MB-231-Luc2 cells. Mice were treated with AZD6244 (25 mg/kg) or vehicle control for 7 consecutive days (beginning at day 14 post cell injection), resulted in a significant attenuation of bioluminescent signals as quantified in (B). Regions of interest (red circles) drawn over the knees (C) and quantified (D) indicated a 10-fold drop in bioluminescent signals at day 21 in response to AZD6244 treatement. Data represented as mean ± SEM (vehicle, N=12; AZD6244, N=6). Asterisks indicate statistical significance (*** p<0.001).

Injection of MDA-MB-231-Luc2 cells into the left ventricle of nude/beige mice invariably resulted in the development of skeletal metastasis, particularly affecting one or both distal femora and/or proximal tibiae. Following injection of MDA-MB-231-Luc2 cells, mice were screened for the development of knee tumor bioluminescence on days 7, 10, 14, 17 and 21 post-IC injection. Mice with nascent bone metastasis were subjected daily treatment of AZD6244, or vehicle control, from days 14 to 21 post-cell injection. AZD6244 treatment dramatically attenuated knee tumor photon emission rates throughout the treatment period (Figure 1A-B), exhibiting an approximately 1 log difference as compared to vehicle-treated controls by day 21 (Figure 1C-D). There was no initial sign of tumor regression, as would be indicated by a net loss of bioluminescence, rather, photon fluxes plateaued throughout the treatment period. Superficially, this finding appeared to be consistent with *in vitro* data showing that MEK inhibition often resulted in a cytostatic effect.

### AZD6244 treatment of bone metastases leads to central loss of tumor cells

**Figure 2:**
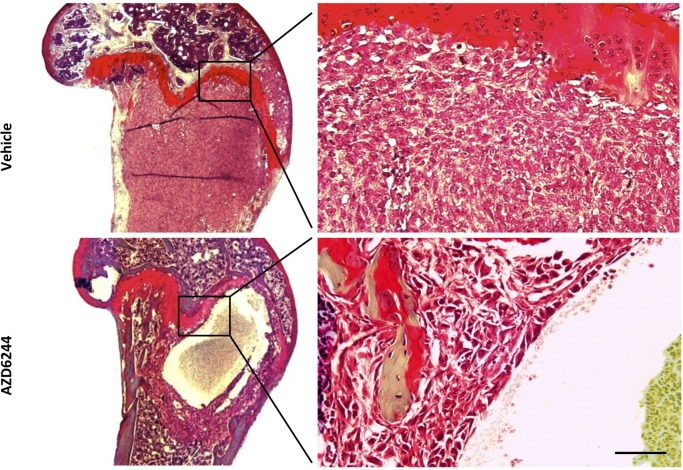
AZD6244 treatment led to the formation of apoptotic cavities within established bone metastases Representative histological sections of tumor-bearing knees were stained by the trichrome method. Vehicle control mice displayed large solid MDA-MB-231-Luc2 tumors that had developed beneath the growth plate, while the tumors in mice treated with AZD6244 had undergone central cavitation. Right panel shows a higher maginification view of the indicated knee region. Scale bar = 100 μm right panel, 1.25 mm left panel.

Intracardiac injection of MDA-MB-231-Luc2 cells led to the development of homogenous osteolytic tumors that eventually filled the medullary cavity of the distal femur and/or proximal tibia. In contrast, bone metastases in mice that had been treated with AZD6244 exhibited large central cyst-like cavities (Figure 2). On the periphery of these cavities there was a layer of tumor cells that was approximately 10 to 20 cells thick and which was well demarcated. This layer appeared viable and even contained some mitotic figures. We hypothesized that the AZD6244-induced central cavities regions likely developed relatively early upon treatment, thus allowing time for dead cells to be cleared by the phagocytic activities of macrophages.

**Figure 3:**
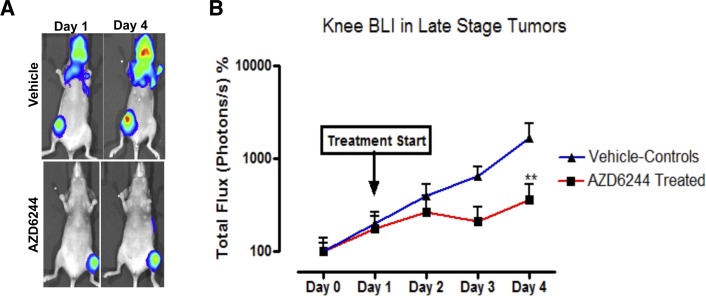
Bioluminescence of fully established metastases was inhibited by AZD6244 treatment A 3 day treatment with AZD6244 (25 mg/kg) was performed on mice with late-stage established metastases (once the total photon fluxes of knees reached an average of 10^7^ photons/s). Bioluminescence imaging (A) showed increased BLI signal in vehicle controls, while the AZD6244-treated mice remained unchanged, as quantified in (B). At the experimental end point (day 4 post-treatment initiation), vehicle-treated controls displayed >10 fold (~1 log) increase in tumor bioluminescence compared to AZD6244 treated mice. Data represented as mean ± SEM (vehicle, N=6; AZD6244, N=5). Asterisks indicate statistical significance (** p<0.01).

**Figure 4:**
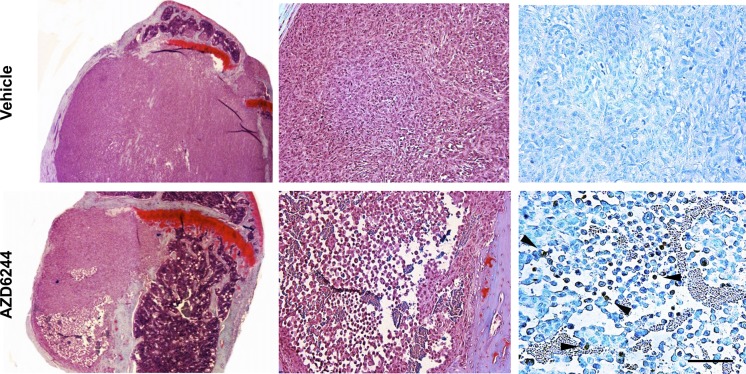
AZD6244 treatment triggered central apoptosis in late stage knee metastases Representative tri-chrome stained images of regions of a late stage MDA-MB-231-Luc2 tibial metastasis demonstrates uniform tumor growth in a vehicle control (upper left; magnified in upper middle panel). In contrast, a large region of cell death is seen at the lower pole of an established metastasis following 3 days of treatment with 25 mg/kg AZD6244 (bottom left; magnified in lower middle panel). TUNEL staining (right panels; arrowheads indicating brown stain with methyl green counterstain) demonstrated the presence of TUNEL-positive cells in the late stage AZD6244 treated metastasis (right lower image). Scale bar = 100 μm right panel; 200 μm middle panel; and 1.25 mm left panel.

The centrally-placed tumor cavities suggested that these were associated with regions of hypoperfusion, raising the possibility that such areas might be particularly sensitive to MEK inhibition and perhaps had undergone cell death in response to AZD6244. To examine this hypothesis further, a short (3 day) treatment regimen with AZD6244 was carried out on large late-stage knee metastases, and tumor viability was monitored via photon flux measurements and BLI (Figure 3A). Following 2 days of treatment with AZD6244 there was a decrease in knee tumor bioluminescence, which was subsequently followed by an increase in photon emission on the last experimental day. However, there was still a significant overall reduction of tumor BLI, as compared to vehicle-treated control mice (Figure 3B). These results showed that even short-term treatment with AZD6244 led to an inhibitory effect on the growth and/or survival of MDA-MB-231-Luc2 bone metastases. We found that in contrast to controls, AZD6244-treated tumors had developed large regions of cell death within the knee metastases (Figure 4), with TUNEL staining revealing the presence of apoptotic cells within the central areas of the metastases. It was therefore possible that this process accounted for the genesis of the large empty cavities observed in tumors when these were treated with AZD6244 from days 14 to 21 (Figure 2).

### MEK inhibition sensitizes MDA-MB-231 cells in low serum conditions

**Figure 5:**
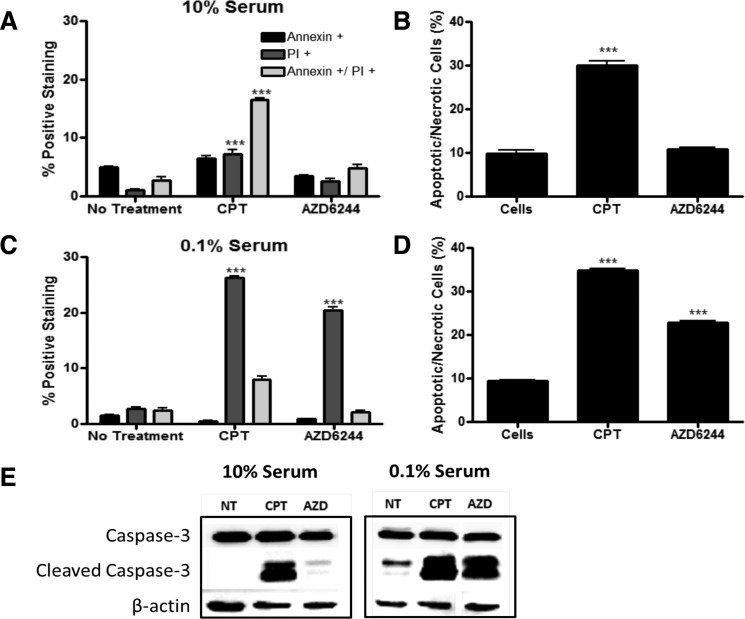
AZD6244 treatment induced apoptosis under low serum conditions Apoptosis was measured by Annexin V-FITC/propidium iodide assay. MDA-MB-231-Luc2 cells were plated in 10% or in 0.1% serum and treated with either 2.5 μm AZD6244, 2.5 μm camptothecin (CPT), or no treatment for 48 hrs prior to imaging. CPT treated cells showed increased cell death and apoptosis in full serum conditions (A and B). In contrast, low serum resulted in increased cell death in both CPT and AZD6244 treated groups (C and D). (E) Western blotting of MDA-MB-231-Luc2 cells exposed to AZD6244 under low serum conditions resulted in a dramatic increase of cleaved caspase-3 protein levels, relative to 10% serum conditions (N=3). Data represented as the mean ± SEM (N=3). Asterisks indicate statistical significance (*** p<0.001).

**Figure 6:**
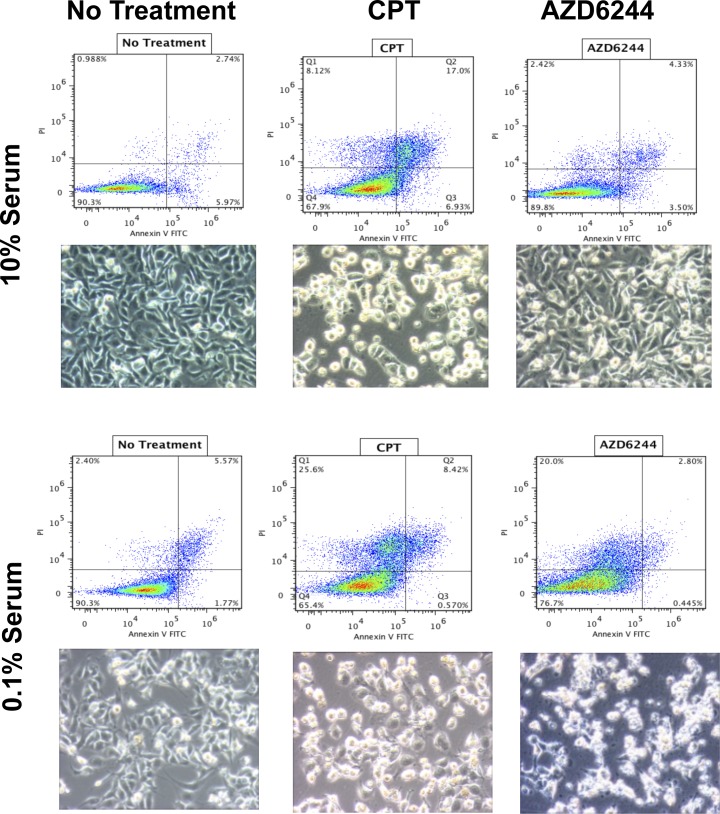
AZD6244 treatment resulted in increased apoptosis and altered cell morphology in low serum Annexin V-FITC/PI FACS plots and cell culture images after 48 hours in either 10% serum; upper panels) or low serum (0.1%); lower panels), and treated with either 2.5 μm AZD6244, 2.5 μm Camptothecin (CPT), or no treatment. CPT-treated cells had increased cell death and apoptosis in both serum conditions as indicated by the Annexin-V/PI double-stained cells in the FACS plots and the cellular morphology. However, a significant increase in cell death (Annexin-V/PI double-stained cells) or rounded-up morphology in the AZD6244 treated cells was seen only under low serum conditions.

In order to investigate the possibility that MDA-MB-231-Luc2 cells may be sensitive to MEK inhibition when subjected to a pro-apoptotic stimulus that plausibly reflected *in vivo* nutrient and/or trophic factor deprivation, MDA-MB-231 cells were grown in 10% FBS and 0.1% FBS. We found that MDA-MB-231-Luc2 cells grown in 10% FBS media and exposed to AZD6244 demonstrated no significant increase in Annexin V-FITC and PI staining (Figure 5A-B), in contrast, cell grown in 0.1% FBS showed considerably increased cell death in response to AZD6244 (Figure 5C-D, and Figure 6). Camptothecin, used as a positive control for apoptosis induction, led to cytotoxicity in both 10% and 0.1% FBS, generating approximately 30%, and 35%, positively-stained cells for Annexin V-FITC, and/or PI, respectively. Immunoblotting showed that in 10% FBS, camptothecin, but not AZD6244 treatment, resulted in elevated levels of cleaved caspase-3. In contrast, in 0.1% FBS, a dramatic increase in cleaved caspase-3 levels was evident in response to AZD6244 (Figure 5E). These findings demonstrated that in the absence of adequate concentrations of serum-derived factors, MDA-MB-231-Luc2 cells became sensitive to AZD6244, and underwent apoptosis. This result provided a potential mechanism for the development of central apoptotic cavity development in the AZD6244-treated bone metastases.

## DISCUSSION

Cancer cells have evolved mechanisms to perpetuate their proliferation and survival, even in the face of toxic stimuli. For example, chemotherapeutic compounds, such as doxorubicin or docetaxel can lead to RAS pathway activation, and this may contribute to the development of drug resistance [17]. Nutrient deprivation and hypoxia, resulting from abnormalities of the tumor microvasculature also constitute toxic stimuli requiring adaptation by cancer cells. The pattern of cell death within the AZD6244-treated metastases, suggested that the RAS-MEK pathway was providing a critical survival mechanism under low nutrient and/or hypoxic conditions likely extant within the centers of the bone tumors. This may have been mirrored by the *in vitro* apoptotic response of the MDA-MB-231-Luc2 cells to the combination of low serum plus AZD6244.

AZD6244 treatment (on days 14 to 21) of bone metastases demonstrated that this agent was able to slow tumor growth as reflected by the decrease in bioluminescence signals as compared to controls. Unexpectedly, histological examination of the AZD6244-treated bone metastases revealed the presence of a single central fluid filled cyst-like space that occupied a large volume within these lesions. These cystic spaces were enveloped by a viable rim of proliferating cells that accounted for the residual bioluminescence that was detected in the AZD6244 treated tumors. Hypothesizing that cystic spaces were the result of MDA-MB-231-Luc2 cell death with subsequent clearance of cell corpses and other debris by macrophages [18], we allowed bone metastases to fully develop before treating them with AZD6244 for only 3 days. This strategy enabled us to capture early drug-induced events, prior to clearance of dead cells by the abundance of phagocytes that are normally present within these metastases (data not shown). Indeed, this experiment demonstrated that AZD6244 treatment led to the development of large centrally located regions of apoptosis within the bone metastases.

The development of central zones of apoptosis in response to AZD6244 was intriguing, and suggested that intra-tumoral heterogeneity was responsible for this phenomenon. It was plausible that the activated *RAS* allele within MDA-MB-231-Luc2 cells was providing survival signals to tumor cells growing in regions that were being subjected to micro-environmental stress. For example, the abnormalities of the solid tumor microvasculature that result in central regions of nutrient and oxygen deprivation suggested an explanation for why AZD6244 had produced central cell death within the bone metastases. Perhaps consistent with this idea, we found that MDA-MB-231-Luc2 cells underwent apoptosis in response to serum deprivation when simultaneously exposed to AZD6244 *in vitro*. This suggested that MEK inhibition sensitized the cells to the pro-apoptotic stimulus of serum deprivation. Since serum contains a complex mixture of trophic factors, low concentrations of serum might plausibly mirror reduced nutrient availability within the central regions of rapidly growing tumors. Indeed, the RAS-MEK pathway may be important in helping tumor cells resist stressors [19]. From a therapeutic perspective, it may be possible to augment the effects of MEK inhibition by interfering with cancer cell energy production, for example, via the use of agents that diminish glycolysis [20]. Alternatively, AZD6244 treatment might be able to augment the apoptotic responses of activated-RAS expressing tumors to cytotoxic agents or radiation therapy.

Under low nutrient conditions, the mutant p53 in MDA-MB-231 cells was previously shown to be stabilized by phospholipase D (PLD), and this was proposed to be under control of the Ras-MAPK pathway [19]. Interestingly, it was also shown that inhibition of PLD activity resulted in the de-stabilization of mutant p53, leading to significant apoptosis following serum deprivation [19]. There is evidence that mutant p53 can exert dominant-negative or gain-of-function properties [21]. For example, genome-wide approaches have determined that mutant p53, but not wild-type p53, regulates the transcription of EGFR and c-MYC [22]. In fact, the mutant p53 effect may contribute to EGFR expression in MDA-MB-231 cells, as well as in a significant portion of aggressive breast cancers associated with the ‘triple-negative’ profile [22]. Furthermore, a positive relationship has been proposed whereby phosphatidic acid (PA), a product of PLD, results in increased RAS-MAPK pathway activity, and where EGFR signalling via RAS-MAPK activates PLD [23]. Therefore, the concerted effects of mutant p53, PLD and the RAS-MAPK pathway may act to suppress cell death during nutrient-deprivation and exposure to other stressors. In addition, MAPK-induced phosphorylation of the pro-apoptotic factor BIM induces its proteasomal degradation, thereby impeding the induction of apoptosis [24]. In contrast, inhibition of MEK would be predicted to promote the pro-apoptotic effect of BIM.

Although MEK inhibition may blunt the ability of tumor cells to respond to a variety of pro-apoptotic stimuli, a potential stumbling block lies in the finding that elevated levels of BCL-2 appear to have the capacity to counteract the increases in BIM brought about by MEK inhibition, thus inhibiting the pro-apoptotic function of BIM [24]. Therefore, simultaneous inhibition of the MEK-MAPK axis and BCL-2 could represent a way to effectively promote BIM-induced cancer cell apoptosis. In fact, it was shown that the cytostatic effects MEK inhibition could be converted into cytotoxicity with resulting long-term regression of tumors caused by cell lines having a constitutively activated *BRAF* allele [25]. These results and those reported herein, would provide impetus to assessing the effectiveness of combining MEK and BCL-2 inhibition as an anti-cancer treatment.

## MATERIALS AND METHODS

### Chemicals and MDA-MB-231-luc2 cells

AZD6244 was purchased from Axon Medchem (Netherlands), camptothecin and MTT were from Sigma, and cell culture reagents from Invitrogen. A polyclonal population of MDA-MB-231 cells stably expressing an EGFP-Luciferase 2 (Luc2) fusion protein (designated herein as MDA-MB-231-Luc2) was generated and maintained as described previously [13]. These cells were confirmed to be free of pathogenic murine viruses and *Mycoplasma spp.* by PCR testing (Charles River Laboratories).

### Apoptosis assay

An annexin V-FITC apoptosis detection kit (BD Pharmingen) was used. MDA-MB-231-Luc2 (10^5^ cells/well) seeded and incubated for 24 hrs, then media was replaced with fresh media containing either 10% or 0.1% FBS followed by 24 hrs of incubation. AZD6244 was added into respective wells and all the cells were incubated for a further 48 hrs. Non-adherent cells and adherent cells (using 1x Versene) were harvested, washed twice with PBS, and once with 1x binding buffer. Cells were stained with 5 μl of Annexin V-FITC in the dark for 15 min and PI was added to the cell suspension prior to being acquired and analyzed by flow cytometry.

### Immunoblotting

Ice-cold RIPA lysis buffer containing a protease and phosphatase inhibitor cocktail (Roche, IN) was used to lyse the cells. Following 30 min in RIPA buffer solution, lysates were centrifuged at 13,000 rpm for 15 minutes. Protein concentrations were determined using Bio-Rad protein dye reagent. Whole cell lysates (30 μg per lane) were then loaded, electrophoresed, and transferred to PVDF membrane (Millipore) using a semi-dry transfer method. Membranes were blocked with 5% skim milk in 1x TBS with 0.1% Tween-20, and probed with primary antibodies (Cell Signalling) in 1% skim milk in 1x TBS with 0.1% Tween-20 at 4 ^o^C overnight. The following day, membranes were probed with the corresponding secondary antibodies and detected using the Immobilon chemiluminescent HRP substrate (Millipore).

### Animals

Female 5-6 week old, NIH-III (*nu/nu; beige/beige*) mice were purchased from Charles River Canada. All animal studies were performed in compliance with Canadian Council of Animal Care guidelines, and with ethics approval from the University of Calgary Animal Care Committee.

### Metastasis model and Bioluminescence imaging (BLI)

To generate metastasis MDA-MB-231-Luc2 cells (2x 10^5^ per mouse) were injected into the left ventricle as previously described [26]. The progression of metastases was monitored by sequential BLI (days 7, 10, 14, 17, 21), and mice were sacrificed on day 21, since after that time, we have found that mice frequent need to be sacrificed owing to the morbidity caused by large tumor burdens.

Bioluminescence imaging was performed as previously described [26]. Mice generated metastases in both legs by day 14; the knees with the highest bioluminescence signals were followed for the remainder of the experiment and used for data analysis in order to reduce variability between treatment groups.

### Drug treatment

Mice with BLI evidence of knee metastases by day 14 post intra-cardiac cell injection were randomly assigned into either experimental, or vehicle control groups, and were then given AZD6244 (25 mg/kg), or vehicle, in 5 ml/kg of 50 mM 2-(hydroxypropyl)-β-cyclodextrin, via daily oral gavage for 7 consecutive days. In contrast, the 3-day treatment regimen was commenced once total photon fluxes in one or both knees had reached an average of 10^7^photons/s, representing a relatively advanced stage of bone metastasis growth.

### Histopathology and immunohistochemistry

Upon sacrifice, hind limbs (femurs and tibiae) were harvested and fixed in 4% paraformaldehyde (PFA) for 24 hrs. The limbs were then placed into fresh 4% PFA for a period of 7 days, followed by 14 days of decalcification in 14% EDTA. Knees were sectioned at 6 μm for immunohistochemistry and histological staining with either Masson's trichrome or with haematoxylin and eosin (H&E). For TUNEL staining, the ApopTag Plus Apoptosis Detection Kit (Millipore, Temecula, CA) was used.

### Statistics

Data were plotted as mean ±SEM and statistical analyses were performed using one-way or two-way ANOVA, with Bonferonni post-tests to determine statistical significance, using Graphpad Prism 4.03 software.
